# Aberrant Calcium Signals in Reactive Astrocytes: A Key Process in Neurological Disorders

**DOI:** 10.3390/ijms20040996

**Published:** 2019-02-25

**Authors:** Eiji Shigetomi, Kozo Saito, Fumikazu Sano, Schuichi Koizumi

**Affiliations:** 1 Department of Neuropharmacology, Interdisciplinary Graduate School of Medicine, University of Yamanashi Chuo, Yamanashi 409-3898, Japan; eshigetomi@yamanashi.ac.jp (E.S.); saitok@koto.kpu-m.ac.jp (K.S.); fsano@yamanashi.ac.jp (F.S.); 2 Department of Neurology, Graduate School of Medical Science, Kyoto Prefectural University of Medicine, Kyoto 602-8566, Japan; 3 Department of Pediatrics, Interdisciplinary Graduate School of Medicine, University of Yamanashi, Chuo, Yamanashi 409-3898, Japan

**Keywords:** reactive astrocytes, calcium signals, epilepsy, Alexander disease

## Abstract

Astrocytes are abundant cells in the brain that regulate multiple aspects of neural tissue homeostasis by providing structural and metabolic support to neurons, maintaining synaptic environments and regulating blood flow. Recent evidence indicates that astrocytes also actively participate in brain functions and play a key role in brain disease by responding to neuronal activities and brain insults. Astrocytes become reactive in response to injury and inflammation, which is typically described as hypertrophy with increased expression of glial fibrillary acidic protein (GFAP). Reactive astrocytes are frequently found in many neurological disorders and are a hallmark of brain disease. Furthermore, reactive astrocytes may drive the initiation and progression of disease processes. Recent improvements in the methods to visualize the activity of reactive astrocytes in situ and in vivo have helped elucidate their functions. Ca^2+^ signals in reactive astrocytes are closely related to multiple aspects of disease and can be a good indicator of disease severity/state. In this review, we summarize recent findings concerning reactive astrocyte Ca^2+^ signals. We discuss the molecular mechanisms underlying aberrant Ca^2+^ signals in reactive astrocytes and the functional significance of aberrant Ca^2+^ signals in neurological disorders.

## 1. Introduction

Astrocytes constitute approximately 30% of the cells of the brain and occupy non-overlapping spatial domains in the central nervous system. Astrocytes not only provide structural, metabolic and homeostatic support for neurons but also actively participate in brain functions [1,2]. Astrocytes do not fire action potentials but are excitable with respect to intracellular signaling. Intracellular ions (e.g., Ca^2+^, Na^+^) and second messengers (e.g., cAMP) of astrocytes change dynamically in space and time in response to stimuli. Recent improvements in methodologies to visualize changes in signaling molecules have revealed novel functions of astrocytes in neuronal circuits [3,4].

Ca^2+^ signals have been extensively studied and well characterized since they were discovered to be fundamental to intracellular signaling and intercellular communication [5]. Ca^2+^ signal in astrocytes may reflect local consumption of energy, circuit activity, and brain states. Numerous proteins that regulate Ca^2+^ transport support the dynamic features of Ca^2+^ signals [6]. The form of Ca^2+^ signals in astrocytes changes acutely and chronically in response to brain insult, such as injury, inflammation or hyperexcitability [7]. Reactive phenotype of astrocytes frequently found in disease shows altered Ca^2+^ signals in response to damaged neuronal tissues. In this review, we focus on recent progress in the understanding of Ca^2+^ signals in reactive astrocytes and we discuss mechanisms underlying aberrant Ca^2+^ signals in reactive astrocytes and their functional significance in disease pathogenesis. We focus on recent findings made in the last five years and, therefore, we do not reference many of the prior studies that are fundamental to our understanding of Ca^2+^ signaling in astrocytes. There are many excellent reviews explaining the roles of astrocytes and reactive astrocytes that should be referred to for further understanding of these topics [1,2,3,4,7,8,9].

## 2. Measurement of Ca^2+^ Signals in Astrocytes

Organic Ca^2+^ indicator dyes have been widely used to measure Ca^2+^ in astrocytes. Bulk-loading of acetoxymethyl (AM) ester forms of indicator dyes is used in many studies because they are easy to load into cells in situ and in vivo and reliably report Ca^2+^ signals. Some AM dyes (e.g., Fluo-4/AM) are preferentially loaded into astrocytes in optimal conditions. However, in theory, these dyes can also enter other cells, therefore, these data must be analyzed with caution. To confirm whether signals are derived from astrocytes, sulforhodamine 101, a red fluorescent dye that selectively labels astrocytes at low concentration is used. Bulk-loading of Ca^2+^ indicator dyes can report Ca^2+^ signals in relatively large regions such as somata and major branches. However, astrocytes are morphologically very complex with many other cellular compartments. Thousands of astrocytic branchlets and leaflets are closely associated with synapses. Most of an astrocyte’s surface area (90–95%) consists of branchlets and leaflets [1]. In these fine structures, Ca^2+^ signals cannot be reliably measured by bulk-loading of a Ca^2+^ indicator. Therefore, most astrocyte territories, especially fine processes that are relevant to interactions with synapses, cannot be observed by this method [10]. Genetically encoded Ca^2+^ indicators (GECIs), such as Yellow Cameleon, GCaMP and GECO, are more suitable for the measurement of Ca^2+^ signals at fine structures because GECIs can be introduced specifically into astrocytes under the control of astrocyte specific promoters. GCaMP3 and membrane tethered GCaMP3 (Lck-GCaMP3) are able to indicate many microdomain Ca^2+^ signals in situ in entire astrocyte territories and significantly improve the detection of Ca^2+^ signals that are missed by bulk-loading of organic Ca^2+^ indicators [11]. Similar microdomain Ca^2+^ signals can be observed in vivo using the latest versions of GECIs [12,13,14,15,16,17]. GECIs can be introduced into astrocytes using adeno-associated viral vectors or transgenic mouse lines. Many transgenic lines are available to drive the expression of GECIs using the Cre-lox and Tet-systems [6,14].

GECIs are superior to organic Ca^2+^ indicator dyes with respect to specificity. Recently, Smith et al. found that high concentrations of organic Ca^2+^ indicators (Fura-2, Fluo-4, and Rhod-2), but not GCaMP3, inhibit Na,K-ATPase. Loading organic Ca^2+^ indicator dyes (or BAPTA) into mice increases intracellular K^+^, probably through reduction of K^+^ uptake. Dye loading also increases extracellular ATP, possibly through dying cells [18]. These findings indicate that data obtained using organic Ca^2+^ indicators should be interpreted with caution.

GECIs can be stably expressed in certain cell types allowing the chronic monitoring of Ca^2+^ activities using two-photon microscopy. However, high expression levels of GECIs can cause cellular damage [19]. Because of their brightness and photostability, GECIs are suitable for wide-field imaging, revealing global Ca^2+^ elevation in astrocytes, which occurs synchronously in many astrocytes throughout the cortex [20]. Noradrenaline, derived from locus coeruleus neurons in response to arousal or startle, causes global Ca^2+^ signals [20,21,22].

## 3. Reactive Astrocytes in Disease

Astrocytes become reactive in response to injury and inflammation. There are at least two distinct categories of reactive astrocytes: hypertrophic reactive astrocytes and scar-forming astrocytes [1]. In this review, we focus on hypertrophic reactive astrocytes, which we term, hereafter, reactive astrocytes. Reactive astrocytes are found in many neurological diseases, such as Alzheimer’s disease (AD), amyotrophic lateral sclerosis (ALS), multiple sclerosis (MS), epilepsy, stroke and traumatic brain injury (TBI) [9,23]. Upregulation of glial fibrillary acidic protein (GFAP) is widely used as a marker of reactive astrocytes. Reactive astrocytes show hypertrophy with thicker processes. Roles of reactive astrocytes can be neuroprotective or neurotoxic, depending on the context. In brain or spinal cord injuries (SCIs), astrocytes become reactive astrocytes with morphological changes. At the site of injury, scar-forming astrocytes form glial scars. Preventing glial scar formation leads to infiltration of circulating immune cells and subsequent neuronal cell damage. Therefore, glial scars provide a barrier around injury sites to protect intact tissues from damage. However, conversely, glial scars are thought to prevent axonal regeneration and functional recovery of neural circuits [24]. Anderson et al. showed that prevention of scar-forming astrocytes do not promote axonal regrowth but reduce stimulated axon regrowth, indicating that scar formation aids rather than prevents recovery of neural circuits from SCI [25]. Thus, glial scars play both beneficial and detrimental roles in axonal regeneration and functional recovery of neural circuits [26].

Zamanian et al. performed transcriptome analysis of reactive astrocytes isolated from inflamed brain after intraperitoneal injection of lipopolysaccharide (LPS), a main component of the outer membrane of gram negative bacteria, or damaged brain following middle cerebral artery occlusion (MCAO) [27]. Differentially expressed genes in LPS-induced reactive astrocytes included genes with potentially detrimental effects, such as *C1r*, *C1s*, *C3* and *C4*, which can cause synapse loss and neuronal damage. Meanwhile, in MCAO-induced reactive astrocytes, differentially expressed genes included neuroprotective genes, such as genes encoding neurotrophic factors, thrombospondins, and cytokines including IL-6. Neurotoxic reactive astrocytes and neuroprotective reactive astrocytes were termed A1 and A2, respectively [28]. These data show the heterogeneity of reactive astrocytes induced by distinct brain insults.

A follow-up study showed that microglia-derived signals (IL-1α, TNF-α and C1q) induce neurotoxic A1 astrocytes. A1 astrocytes release unidentified toxic molecules onto neurons and oligodendrocytes, lose ability to promote neuronal survival, outgrowth and synaptogenesis and impair phagocytosis [28]. Recent evidence shows that astrocytes are heterogeneous with respect to transcriptome, proteome and function; region- and circuit-specific functions have been described [29,30,31]. Therefore, response to injury or inflammation is likely to be distinct depending on the circuits involved and the phenotypes of reactive astrocytes may be more heterogeneous than currently recognized.

Many studies over the past few decades have indicated that reactive astrocytes lose homeostatic functions, including excitatory neurotransmitter uptake, potassium ion uptake, and ion buffering and thus passively contribute to disease pathogenesis [32]. However, recent evidence indicates that reactive astrocytes actively contribute to disease processes even in the presymptomatic phase (see below). A key process in this active role is Ca^2+^ signaling.

## 4. Ca^2+^ Signals in Reactive Astrocytes

In parallel with morphological changes, reactive astrocytes demonstrate dynamic, aberrant Ca^2+^ signals. In most cases, Ca^2+^ signals increase in terms of amplitude, duration and frequency. There is huge variation in the Ca^2+^ dynamics of reactive astrocytes in distinct pathological models, phases (acute or chronic) and regions, indicating diverse underlying mechanisms for aberrant Ca^2+^ signals that are dependent on the conditions.

Astrocytes respond rapidly to injury and hyperexcitability to generate Ca^2+^ signals [33,34,35,36,37,38]. For example, astrocytes rapidly increase their Ca^2+^ in response to hyperexcitability in drug (kainate and pilocarpine)-induced seizure model. In adult mice, simultaneous Ca^2+^ imaging from both neurons and astrocytes using two different colored GECIs revealed that astrocytes are activated earlier than neurons following seizure induced by intraperitoneal administration of kainate. Although the detailed mechanism underlying this astrocyte response is not clear, suppression of Ca^2+^ responses by deletion of inositol-1,4,5 trisphosphate receptor type 2 (IP_3_R2, see below), in which astrocytes lack a major intracellular Ca^2+^ release pathway, resulted in less kainate-induced epileptic activity recorded by electroencephalogram telemetry [38]. This indicates that astrocyte Ca^2+^ signals are proconvulsive, which is consistent with previous reports [36]. In adult mice, 2–3 days after pilocarpine-induced status epilepticus (SE), astrocytes started to show larger Ca^2+^ signals, which may contribute to the delayed loss of neurons because suppression of enhanced Ca^2+^ signals by BAPTA-AM, a membrane permeable Ca^2+^ chelator, which presumably was preferentially loaded into astrocytes in the condition, reduced neuronal damage [39]. Astrocyte Ca^2+^ signals recovered to control levels five days after SE. Interestingly, four weeks after pilocarpine-induced SE, reactive astrocytes showed large Ca^2+^ signals, which may regulate seizure susceptibility in adult mice (Sano et al., University of Yamanashi; unpublished observation). It appears that reactive astrocytes regain aberrant Ca^2+^ activities during the recovery from the damage caused by the initial SE. Thus, the role of Ca^2+^ signals in reactive astrocytes may be distinct during various phases after initial SE (e.g., neuron loss, seizure threshold). Enhanced Ca^2+^ signals in reactive astrocytes are frequently described; however, astrocytes in seizure models do not always show enhanced Ca^2+^ signals. Plata et al. reported that 2-4 weeks after pilocarpine-induced SE in young rats (3–6 weeks old), hippocampal astrocytes showed fewer Ca^2+^ signals [40]. These astrocytes also showed atrophy, but not hypertrophy. Sholl analysis showed reduced complexity of structures in atrophic reactive astrocytes, which may decrease support for synapses from astrocyte processes [41]. Astrocytes can, therefore, be hypertrophic and atrophic in response to hyperexcitable neurons. It is unclear what determines astrocyte phenotypes after SE. The mechanism underlying functional and structural changes in astrocytes in response to hyperexcitability may provide useful information for the etiology of epilepsy.

Aberrant Ca^2+^ signals are preferentially observed in the area where the tissue is strongly affected and where hypertrophic astrocytes are located. For example, in an in vivo adult mouse model of familial AD, reactive astrocytes displayed frequent Ca^2+^ signals near amyloid plaques and Ca^2+^ waves that originated from plaques [42,43]. In an acute stroke model, reactive astrocytes in the ischemic core displayed much larger Ca^2+^ signals in terms of ΔF/F compared with those in the penumbra region [44]. Suppression of such large Ca^2+^ signals reduced the extent of the damaged areas and the number of injured cells. Therefore, these Ca^2+^ signals in reactive astrocytes have a harmful effect in neuronal tissues.

Differences in brain regions and experimental models dramatically affect the Ca^2+^ signals observed. Even in the same ischemia model, the age of animals significantly affects Ca^2+^ signals in reactive astrocytes in the penumbra, where neurons lose the ability to generate spontaneous or evoked electrical activity and astrocytes become reactive. In an MCAO model using adult (3–4-month-old) and aged (18–24-month-old) mice, Fordsmann et al. observed Ca^2+^ activities in both neurons and astrocytes. In adult mice, Ca^2+^ signals in both neurons and astrocytes were suppressed 2–4 h after MCAO, while, in aged mice, Ca^2+^ signals in astrocytes were enhanced and Ca^2+^ in neurons was unchanged. Enhanced Ca^2+^ signals in aged mouse astrocytes were action potential-dependent and occurred through P2 receptor activation, which may be harmful to neurons [45]. These observations clearly show that astrocyte Ca^2+^ dynamics and their roles are distinct at different ages.

Severity of a disease relates to augmentation of aberrant Ca^2+^ signals in reactive astrocytes. In a model of Alexander disease (AxD), a rare neurodegenerative disease caused by autosomal dominant gain of function mutations in *GFAP*, we recently found extraordinarily large Ca^2+^ signals in astrocytes, whose areas were over 300 μm^2^. In contrast, local Ca^2+^ signals (<300 μm^2^) were mainly observed in control mice [46]. We called the large Ca^2+^ signals seen in AxD model mice, aberrant extra-large Ca^2+^ signals (AxCa). Reactive astrocytes in homozygotes showed a higher frequency of AxCa and higher GFAP expression compared with those in hemizygotes. Astrocytes derived from older mice showed higher AxCa frequency and higher GFAP expression in the same genotype. Interestingly, there was a strong positive correlation between AxCa frequency and GFAP expression, a hallmark of disease severity of AxD. Genetic deletion of IP_3_R2 abolished AxCa and reduced GFAP expression in AxD astrocytes, indicating a causal relationship between AxCa frequency and GFAP expression. These data indicate that aberrant Ca^2+^ signals are not just epiphenomena of the disease, but actually partly determine the severity of the disease [46].

Overall, the spatiotemporal dynamics of Ca^2+^ in reactive astrocytes are highly diverse. The frequency and size of Ca^2+^ signals may be good indicators of the phenotype of reactive astrocytes and of disease severity. Ca^2+^ signals should not be interpreted in a binary manner for the downstream signaling of astrocytes [47,48]. Ca^2+^ dynamics, including amplitude, duration, and frequency, may encode information of astrocytes. For example, in situ imaging data indicate that Ca^2+^ oscillations are associated with hypertrophy of astrocytes [49]. Therefore, exploring Ca^2+^ signals in reactive astrocytes provides insight into functional changes.

## 5. Mechanisms of Aberrant Ca^2+^ Signals

In normal physiology, astrocytes display Ca^2+^ signals spontaneously and in response to receptor activation. The most well-defined Ca^2+^ mechanism is Ca^2+^ release from the endoplasmic reticulum (ER) through IP_3_R. There are three isoforms of mammalian IP_3_R. Among them, IP_3_R type2 (IP_3_R2) is well characterized and thought to be a major isoform in astrocytes. Petravicz et al. found that astrocytes in IP_3_R2 knockout mice (IP_3_R2KO) have almost no spontaneous or evoked Ca^2+^ signals [50]. IP_3_R2KO mice have, therefore, been frequently used to remove Ca^2+^ signals from astrocytes. However, IP_3_R2 is not the sole IP_3_R in astrocytes. Recently, IP_3_R1 and IP_3_R3 have also been revealed to contribute to Ca^2+^ signals in astrocytes [51]. Their contribution to Ca^2+^ signals seems to be much smaller than that of IP_3_R2. IP_3_R1 and IP_3_R3 are likely to contribute to locally confined Ca^2+^ signals, but not to spreading Ca^2+^ signals. In contrast, IP_3_R2 contributes to spatially spreading Ca^2+^ signals in astrocytes. The IP_3_R2KO mouse is a useful tool to analyze global Ca^2+^ signals in astrocytes in vivo. However, data obtained from IP_3_R2KO mice should be carefully interpreted. Firstly, a substantial amount of Ca^2+^ signal remains in astrocytic processes in IP_3_R2KO mice [14,52]. Secondly, Ca^2+^ release from the ER is retained in the KO [53], probably through other IP_3_Rs or rynanodine receptors. Thirdly, the conventional IP_3_R2KO, which is widely used in the field, may show increased innate immunity [54], which may complicate data interpretation especially in disease models.

### 5.1. Receptor-Mediated Ca^2+^ Signals

Astrocytes express a plethora of Gq-protein coupled receptors (GqPCRs), activation of which leads to Ca^2+^ release from the ER via IP_3_Rs. Among GqPCRs in astrocytes, metabotropic glutamate receptor 5 (mGluR5) has attracted much attention because it is a receptor for the major excitatory synaptic neurotransmitter, glutamate, and is thought to be central to gliotransmitter release. mGluR5 expression is mainly observed in developmental stages and its expression is dramatically decreased in the adult. Expression of mGluR5 in astrocytes is negligible in the adult brain [55]. Instead of mGluR5, adult astrocytes express mGluR2/3 to receive neuronal information [56]. Interestingly, mGluR5 expression reemerges in reactive astrocytes in pathophysiological conditions such as AD [57,58], epilepsy [59] and neuropathic pain [60].

A few days after pilocarpine-induced SE, cortical astrocytes displayed massive Ca^2+^ signals in vivo, which were inhibited by MPEP, a mGluR5-specific antagonist, indicating that astrocytes express functional mGluR5 after SE [39]. Consistent with this, mGluR5 immunoreactivity was upregulated in reactive astrocytes in a temporal lobe epilepsy rat model [59]. Umpierre et al. confirmed the reemergence of mGluR5 expression in reactive astrocytes in an SE model using conditional mGluR5KO mice [61]. The authors selectively deleted mGluR5 expression from astrocytes using the Cre-lox system with Aldh1l1-CreER^T2^ or GFAP-CreER^T2^. In these animals, only a small proportion of astrocytes responded to DHPG, a mGluR5 agonist, after SE, while in control mice, most astrocytes responded to the mGluR5 agonist. The authors demonstrated slow clearance of glutamate released from synapses in conditional mGluR5 KO mice after SE, indicating that mGluR5 upregulation in astrocytes after SE may be beneficial for glutamate uptake. In neuropathic pain model, astrocytes in the primary somatosensory (S1) cortex became reactive and showed robust mGluR5-mediated Ca^2+^ signals in vivo, 3–6 days after peripheral nerve injury [60]. Interestingly, the reemergence of mGluR5 in astrocytes requires a few days later after initial insults [39,60,61]. The mechanism of its upregulation in vivo is currently unknown. Signaling molecules, such as Aβ and neurotrophic factors, induce mGluR5 expression in cultured astrocytes [58,62].

mGluR5 signaling in astrocytes is altered by not only mGluR5 gene expression but also the protein interaction with a scaffold protein, Homer1. Homer1a, a splice variant of Homer1, was upregulated in reactive astrocytes, causing a reduction of mGluR5-mediated Ca^2+^ signals and its downstream mechanisms [63].

The purinergic receptor, P2Y1, is another important GqPCR in reactive astrocytes. In contrast to the mGluR5 receptor, the P2Y1 receptor is expressed in astrocytes throughout life. Endogenous ligands for the P2Y1 receptor are ATP and ADP. ATP can be released from various brain cell in the brain through various mechanisms, such as channels, transporters and exocytosis [64]. P2Y1 receptor-mediated Ca^2+^ signals occur in response to increased neuronal excitability or even spontaneously. Overexpression of the P2Y1 receptor specifically in astrocytes preferentially increased Ca^2+^ wave-like signals rather than microdomain Ca^2+^ signals [65]. This is consistent with reports showing that P2Y1 receptors mediate Ca^2+^ waves [35,64]. The P2Y1 receptor is upregulated in pathophysiology, including in AD, stroke and epilepsy [42,66,67]. In familial AD model mice, ~38% of in vivo cortical astrocytes were defined to be hyperactive displaying a high frequency of Ca^2+^ signals. Hyperactive astrocytes were found near amyloid plaques. Ca^2+^ signals in hyperactive astrocytes were independent from action potential and inhibited by MRS 2179, a P2Y1 receptor antagonist, indicating that ATP or ADP release near plaques contributed to the signals [42]. Carbenoxolone reduced the number of hyperactive astrocytes, indicating that ATP may be released via hemichannels. MPEP did not affect Ca^2+^ signals in either astrocytes or neurons, indicating that mGluR5 does not contribute to the hyperactivity [42], in contrast to immunohistochemical data showing upregulation of mGluR5 in reactive astrocytes near plaques [57,58]. A follow-up report by the same group showed that pharmacological blockade of P2Y1 receptors chronically ameliorated synaptic deficits and restored spatial learning and memory in the mice. Deletion of IP_3_R2, a downstream molecule in the P2Y1 receptor pathway, from the AD model also improved spatial memory [68]. This series of studies indicates that the P2Y1 receptor mediates Ca^2+^ signals and may be a therapeutic target to treat some symptoms of AD.

In kindled rats, reactive astrocytes in the hippocampus showed longer duration Ca^2+^ signals, termed slow transients, which were not observed in control rats [66]. Similar to Ca^2+^ signals in the AD model, slow transients were independent of action potentials and mediated by the P2Y1 receptor because slow transients were reduced by MRS 2179 but not by MPEP. The ligands to activate P2Y1 receptors may be released through pannnexin-1 channels [69]. Overall, the P2Y1 receptor is upregulated in reactive astrocytes. ATP or ADP seem to be released via hemichannels/pannexins. What triggers the release of ATP? Nikolic et al. found that a puff of TNF-α, a cytokine that regulates synapse functions and cell death, caused Ca^2+^ elevation in the astrocyte molecular layer of the dentate gyrus [70]. This elevation was reduced by MRS 2179, indicating that autocrine or paracrine release of ATP activated P2Y1 receptors to cause TNF-α-induced Ca^2+^ elevation. This indicates that TNF-α can trigger ATP release from astrocytes via unknown mechanism. TNFα-activated P2Y1 receptors contribute to enhancement of excitatory synaptic transmission onto granule neurons in seizure [70]. It would be interesting to determine if enhanced Ca^2+^ signals in reactive astrocytes in a seizure model are actually mediated by TNF-α-P2Y1 receptor signaling.

GABA released from neurons activates astrocytic GABA_B_ receptors resulting in Ca^2+^ signals [71,72]. Optogenetic activation of interneurons caused interneuron subtype-specific Ca^2+^ elevation in astrocytes [72]. In pathological conditions, GABA_B_ receptors in astrocytes contribute to Ca^2+^ oscillations after cortical spreading depression (CSD) [73] or stroke [44], resulting in increased neuronal excitability and damage. In a CSD model, neither mGluR5 nor P2 receptors contributed to the Ca^2+^ oscillation, but a GABA_B_ antagonist reduced the oscillation. This indicates that GABA may be preferentially released in the phase when astrocytes display Ca^2+^ oscillations in the CSD model [73].

The adenosine A_2A_ receptor is a Gs-protein coupled receptor. There is no clear evidence to show that activation of the A_2A_ receptor can lead to Ca^2+^ signals in astrocytes in vivo. Chemogenetic activation in astrocytes via Gs-DREADD causes Ca^2+^ signals [29]. The A_2A_ receptor is upregulated in human AD tissues and upregulation of A_2A_ in AD model mice contributes to reduce memory performance [74].

### 5.2. Transmembrane Ca^2+^ Pathways

Ca^2+^ influx through the plasma membrane is also relevant to Ca^2+^ signals in astrocytes. Srinivasan et al. showed that processes but not soma of cortical astrocytes in behaving mice display Ca^2+^ signals that are independent of IP_3_R2 and GqPCR activation [14]. The authors also found that Ca^2+^ influx contributed to Ca^2+^ signals in astrocytes because nominally Ca^2+^-free conditions strongly reduced Ca^2+^ signals at processes. Rungta et al. also found that Ca^2+^ signals at fine astrocyte processes occur through Ca^2+^ influx pathways in the hippocampus [52]. Many types of Ca^2+^-permeable channels are thought to be expressed in astrocytes, such as AMPA receptors, NMDA receptors, α7 nicotinic receptor, P2X1 receptor, P2X7 receptor, TRPA1, TRPCs and TRPV4 [6,8]. The TPRA1 channel partly contributes to microdomain Ca^2+^ signals in astrocytes, which can be detected by Lck-GCaMP, a membrane-targeted GECI [15,52,75].

In contrast to aged mice, young (one-month-old) familial AD mice do not show reactive astrocytes, which was assessed by GFAP expression. This early phase should be considered a presymptomatic phase. However, approximately 20% of astrocytes display Ca^2+^ frequency hyperactivity in situ. This hyperactivity is reduced by HC030031, a TRPA1 channel blocker, indicating that TRPA1 contributes to aberrant Ca^2+^ signals in an early phase of the disease [76]. The Aβ oligomer triggers Ca^2+^ signals in naïve mice. Therefore, production/accumulation of Aβ oligomer may be a key process for such astrocyte hyperactivity in the AD model. The TRPA1-mediatd Ca^2+^ signal of astrocytes in the AD model contributes to increased excitatory synaptic transmission [76]. TRPA1 in astrocytes also contributes to the expression of proinflammatory cytokine genes in the AD model [77]. Furthermore, Aβ_1-42_ caused Ca^2+^ elevation in astrocytes through α7 nicotinic receptor causing gliotransmission [78]. Thus, multiple Ca^2+^ flux pathway may contribute to AD disease process.

TRPC4 channels in astrocytes were upregulated in MeCP2-deficient astrocytes, also termed Rett syndrome (RTT) astrocytes. Ca^2+^ content in the ER of RTT astrocytes was highly elevated (i.e., Ca^2+^ overload), resulting in highly frequent Ca^2+^ signals that occur spontaneously [79]. Knockdown of TRPC4 in RTT astrocytes ameliorated the Ca^2+^ overload in the ER and resulted in fewer abnormal Ca^2+^ signals in RTT astrocytes. TRPC4-mediated abnormal Ca^2+^ signals in RTT astrocytes trigger astrocytic glutamate release to activate extrasynaptic NMDA receptors in neurons leading to network hyperexcitability in RTT mice [79].

Ischemic stroke causes irreversible damage to neuronal tissues. However, mild ischemia that does not cause severe symptoms in animals makes these tissues/animals more tolerant to subsequent, more severe ischemia. This phenomenon is called ischemic tolerance. Hirayama et al. found that short-term MCAO, which triggers ischemic tolerance, induced reactive astrocytes, while impairment of reactive astrocytes abolished the ischemic tolerance. The purinergic P2X7 receptor, an ATP-gated Ca^2+^ permeable channel, is selectively upregulated in these astrocytes and is essential for astrocyte-mediated ischemic tolerance [80]. P2X7 receptor upregulation in reactive astrocytes leads to HIF1α induction for long-lasting neuroprotection [81]. This phenotypic change following mild ischemia is consistent with the idea that A2 astrocytes induced by MCAO upregulate neuroprotective genes [27].

### 5.3. Ca^2+^ Release from Mitochondria

Agarwal et al. found that a substantial portion (~55%) of microdomain Ca^2+^ signals occurred via Ca^2+^ efflux from mitochondria via mitochondrial permeability transition pores (mPTPs). Mitochondrial Ca^2+^ efflux was increased by reactive oxygen species production. In an ALS model, mutations in the mitochondrial enzyme gene, superoxide dismutase 1, caused more microdomain Ca^2+^ signals, indicating that mitochondrial stress causes mPTP opening [15]. Ca^2+^ signals through mPTP openings are suggested to relate to the metabolic demands of neurons. Removal of external Ca^2+^ reduced microdomain Ca^2+^ signals by 52–90% [14,52]. This indicates that mitochondrial Ca^2+^ may be immediately replenished by Ca^2+^ from the extracellular space.

### 5.4. Other Mechanisms

As mentioned above, we found aberrant Ca^2+^ signals in astrocytes termed AxCa in an AxD model. Pharmacological profiles of Ca^2+^ suggest that AxCa is independent of action potentials, P2 receptors, mGluR5, mGluR2/3, adrenergic α1 receptors and A_2A_ receptors. The mechanisms underlying AxCa action are still not clear, but our data indicate that Ca^2+^ handling at the ER may be important [46]. Consistently, Jones et al. recently found that disrupted ER distribution and abnormal Ca^2+^ transport by *GFAP* mutation in AxD patients [82]. Accordingly, Ca^2+^ release through IP_3_R2 is a major pathway for the generation of AxCa [46].

Similarly, cultured Down syndrome (DS) astrocytes, generated from human DS stem cells, show aberrant Ca^2+^ signals. These Ca^2+^ signals are also independent of GPCRs, such as mGluR5, P2 receptors and adenosine A_1_ receptors, but dependent on IP_3_R2. The S100β gene, which encodes a Ca^2+^ binding protein that is preferentially expressed in astrocytes, is located on human chromosome 21, and is, therefore, overexpressed in DS. S100β causes the aberrant Ca^2+^ signals by acting on intracellular rather than extracellular targets, resulting in suppression of neuronal activities via A_1_ receptors [83].

## 6. What Is the Function of the Aberrant Ca^2+^ Signal in Reactive Astrocytes?

**Figure 1:**
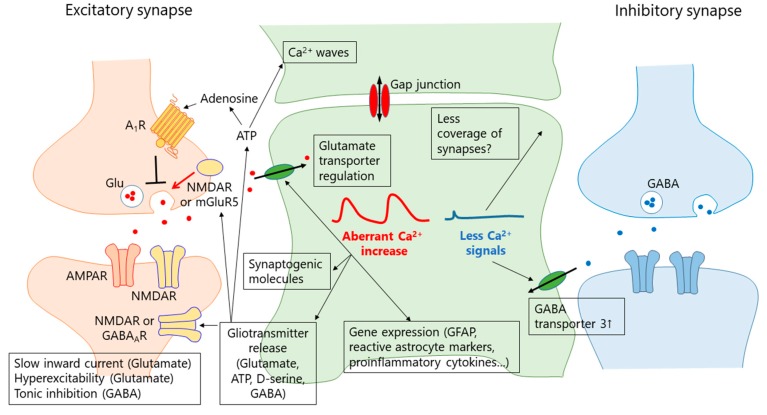
Functional significance of astrocyte Ca^2+^ signals in disease. The cartoon indicates how alteration of astrocyte Ca^2+^ in disease affects excitatory/inhibitory synapses and excitability of neurons.

Ca^2+^ is a ubiquitous second messenger regulating multiple aspects of cellular signaling. There are many mechanisms proposed for the functions of aberrant Ca^2+^ signals in reactive astrocytes that are summarized in Figure 1. Ca^2+^-dependent gliotransmission has attracted much interest because it is a well-known and well-characterized feature of astrocytes, although its relevance and mechanism are still under debate [84,85].

### 6.1. Gliotransmission

Astrocytes can release gliotransmitters, such as glutamate, ATP, D-serine and GABA in a Ca^2+^-dependent manner. Glutamate derived from astrocytes activates NMDA receptor on neurons in epilepsy [36,39,66,70,86], ischemia [34,87], CSD [73], Alzheimer’s disease [68] and Rett syndrome [79]. Activation of presynaptic NMDA receptors or mGluR5 by glutamate from astrocytes enhances excitatory synaptic transmission [66,70,88,89], while postsynaptic activation of NMDA receptors may lead to hyperexcitablity [34,36,39,73]. Glial-dependent presynaptic NMDA receptor activation is enhanced by TNF-α [90], which contributes to cognitive impairment in experimental autoimmune encephalitis (EAE), an animal model of MS [88]. Thus, NMDA receptor activation presumably triggers increased excitation of networks and neuronal death. One of the important issues in the field is whether or not astrocytes release glutamate in a Ca^2+^-dependent manner [29,84,85,91,92]. The machinery for glutamate release is undefined because astrocytes lack the molecules for vesicular glutamate release [29,93], although exocytosis of glutamate from astrocytes has been proposed. Another important issue is the functional significance of glutamate release from astrocytes. In many cases, slow inward currents (SIC) are recorded as an indicator of glial-derived glutamate release. SICs are thought to be caused by activation of extrasynaptic NMDA receptors in postsynaptic sites via glutamate released from astrocytes. SICs are blocked by antagonists against NMDA receptor containing NR2B subunit such as D-AP5 and Ro 25-6981. Recently, Gomez-Gonzalo et al. found that spontaneous SICs were mediated by a channel sensitive to 4,4’-Diisothiocyano-2,2’-stilbenedisulfonic acid (DIDS), quinine and fluoxetine but not by Ca^2+^-dependent vesicular glutamate release from astrocytes [94]. It has been suggested that glutamate derived from astrocytes, which cause SICs, contributes to many neurological diseases, such as epilepsy [36,39,66], stroke [34,87] and neurodevelopmental disorders [79]. Currently, there is no specific way to inhibit glutamate release mechanism underlying SICs without affecting other cell types/mechanisms. Therefore, the pathophysiological significance of SICs has not been tested directly. These two issues need to be solved to understand the role of Ca^2+^-dependent glutamate release from reactive astrocytes. In addition to a Ca^2+^-dependent mechanism, astrocytes can release glutamate in an intracellular pH-dependent manner. Oxygen glucose deprivation (OGD) reduces intracellular pH to cause glutamate release, which underlies ischemic brain damage [95].

In an APP/PS1 familial AD mouse model, reactive astrocytes in the dentate gyrus upregulate monoamine oxidase B, which contributes to GABA synthesis in reactive astrocytes. This astrocytic GABA is released through BEST1 channels in a Ca^2+^-dependent manner, causing intense tonic GABA currents. A strong inhibitory effect of astrocyte-derived GABA impaired neurotransmitter release, action-potential firing, synaptic plasticity and memory, which may underlie cognitive impairment in AD [96]. In other AD mouse model, tonic GABA inhibition was also enhanced and impaired synaptic plasticity, although GABA was released by the reverse mode of GABA transporters [97]. Increased GABA expression is also found in hypertrophic astrocytes induced by a stab wound brain injury model. Then, increased GABA is considered as a maker for reactive astrocytes [98].

ATP is also released from reactive astrocytes. ATP activates purinergic receptors in reactive astrocytes in an autocrine/paracrine fashion to contribute to Ca^2+^ waves [42,64]. These Ca^2+^ waves propagate to areas remote from the initiation site to transmit the information to synapses and microglia [99], which may cause hyperexcitability/damage and microglial chemotaxis to the injury site [35,64,100]. Many studies suggest that ATP acts on P2Y1 receptors on astrocytes to induce glutamate release in a Ca^2+^-dependent manner [64,66,70,89,90]. P2Y1 receptor-mediated glutamate release could be relevant to pathological conditions because TNF-α, an inflammatory cytokine induced by injury and seizure, is able to enhance P2Y1 receptor-mediated Ca^2+^ signals and gliotransmission [70,90]. In EAE, TNF-α levels are increased. Pathological levels of TNF-α altered synaptic transmission in the dentate gyrus, contributing to memory deficits in EAE [88]. It is not clear whether P2Y1 receptor-mediated Ca^2+^ signals are involved in this TNF-α effect. However, it is intriguing to see whether P2Y1 receptor-mediated Ca^2+^ signals contribute to the memory deficits in EAE. ATP is degraded into adenosine via ecto-nucleotidase, which in turn activates adenosine A_1_ receptors to inhibit neuronal excitability and excitatory synaptic transmission [83]. Thus, ATP derived from astrocytes either excites or inhibits neuronal networks, which depends on the context.

### 6.2. Synapse Remodeling

Developmental stage astrocytes express synaptogenic molecules, such as thrombospondin 1 (TSP1) [101]. TSP1 expression is low in adult mice but upregulated in pathophysiology. In a neuropathic pain model following peripheral nerve injury, TSP1 was upregulated in astrocytes of the S1 cortex through mGluR5-mediated Ca^2+^ signals in astrocytes [60], blockade of which reduced chronic pain. Also, pharmacological inhibition of the TSP1 receptor or TSP1 knockdown reduced chronic pain. Furthermore, TSP1 contributes to synapse remodeling in S1 cortex in neuropathic pain models. Thus, TSP1 upregulation through mGluR5-mediated Ca^2+^ signals in astrocytes cause synaptic rewiring in the S1 circuits by forming novel connections between neurons that underlie neuropathic pain [60,102].

### 6.3. GFAP Upregulation

GFAP is the most established maker for astrocytes and its upregulation is generally considered as a maker for reactive astrocytes. GFAP accumulation/upregulation is found in many neurological diseases, including AD [103]. Aberrant Ca^2+^ signal is essential for GFAP upregulation in AxD [46], TBI [37] and photothrombosis [104], since deletion of aberrant Ca^2+^ signals strongly suppressed the GFAP accumulation/upregulation. Thus, Ca^2+^ signals in astrocytes seem to be common events to induce GFAP upregulation. Because chronic treatment with a P2Y1 receptor antagonist reduced astrocyte Ca^2+^ signals in AD model mice but not GFAP upregulation, astrocyte Ca^2+^ signals may be relevant to induction of GFAP expression rather than maintenance of the expression.

In addition to GFAP upregulation, IP_3_R2-mediated Ca^2+^ signals regulate gene expression of other genes. AxCa regulate *Lcn2*, a pan reactive astrocyte maker, and *C3*, an A1 (neurotoxic) astrocyte marker [46]. Kanemaru et al. show that TBI-induced Ca^2+^ signals via IP_3_R2 inhibit the translational repressor, Pum2, to upregulate N-cadherin expression, which prevents infiltration of leukocytes and is neuroprotective [37]. Thus, IP_3_R2-mediated Ca^2+^ signals are essential for GFAP upregulation in pathological conditions.

### 6.4. Neuronal Damage

Infarct volume following MCAO or photothrombosis is significantly smaller in IP_3_R2KO mice compared with controls [34,87,104]. IP_3_R2 is upregulated in the penumbra by photothrombosis [54], which may contribute to aberrant Ca^2+^ signals in astrocytes of this region. Rakers et al. found that IP_3_R2 contributes to peri-infarct depolarization (PID), which is thought to enhance neurodegeneration and expand infarct size. The authors imaged Ca^2+^ in neurons and astrocytes in vivo after permanent MCAO and found PID-related Ca^2+^ elevations in both cell types. Ca^2+^ elevations in astrocytes were significantly reduced in IP_3_R2KO. Interestingly, PID-related Ca^2+^ elevations in neurons were also reduced in IP_3_R2KO, suggesting that astrocytic Ca^2+^ waves enhance neuronal Ca^2+^ elevations in PID. Astrocyte Ca^2+^ elevation is positively correlated with extracellular glutamate increase [87]. Lack of IP_3_R2 shortened both the duration of astrocyte Ca^2+^ elevation and duration of glutamate increase. Thus, glutamate levels are further increased by glutamate derived from Ca^2+^-elevated astrocytes, which contributes to excitotoxicity following ischemia. Similarly, Dong et al. found that in OGD, an in vitro model of ischemia, Ca^2+^ waves were induced in astrocytes in an IP_3_R2-dependent manner. SICs induced by OGD were also reduced in IP_3_R2KO mice, indicating that glutamate release from astrocytes may be augmented [34]. Thus, ischemia causes extracellular glutamate levels to rise by Ca^2+^ signals via IP_3_R2 in astrocytes, which contribute to glutamate toxicity through the activation of extrasynaptic NMDA receptors. These findings indicate that augmented IP_3_R2-mediated Ca^2+^ signals exacerbate damage by ischemia probably through elevation of extracellular glutamate [34,87]. These lines of evidence indicate that IP_3_R2-mediated Ca^2+^ signals can be a therapeutic target to protect neurons from ischemic damage. A transmembrane pathway through TRPV4 also contributes to astrocyte Ca^2+^ elevation following permanent MCAO. Although its contribution is smaller than that of IP_3_R2-mediated Ca^2+^ signals [105], recent evidence indicates that TRPV4 activation is a key determinant in brain edema induced by ischemia [106].

## 7. Role of Reduced Astrocyte Ca^2+^ Signals in Disease

Generally, reactive astrocytes display augmented Ca^2+^ signals; however, Ca^2+^ signals in astrocytes can also be reduced in pathophysiology [40,107]. There are several ways to reduce Ca^2+^ signals experimentally. Firstly, IP_3_R2KO mice have strongly reduced spontaneous and evoked Ca^2+^ signals in the cytosol, as described above [50]. Secondary, the “IP_3_ sponge”, an IP_3_ absorber, is another way to reduce IP_3_-mediated Ca^2+^ signals. Selective introduction of an IP_3_ sponge into astrocytes leads to reduced coverage of synapses by astrocytes and facilitated spillover of glutamate from synapses [41]. Thirdly, IP_3_ 5-phosphatase, an IP_3_ hydrolyzing enzyme, is a useful tool to suppress IP_3_-mediated Ca^2+^ signals in astrocytes [108]. Fourthly, Yu et al. showed that overexpression of human PMCA2w/b (hPMCA2w/b), which constitutively excludes Ca^2+^ from the cytoplasm in astrocytes, strongly reduces spontaneous and evoked Ca^2+^ signals [109]. Overexpression of hPMCA2w/b in striatal astrocytes caused an increase in self-grooming behavior. Detailed molecular and functional analysis of astrocytes and medium spiny neurons (MSNs) in the striatum revealed altered MSN activity resulting from increased expression of GABA transporter 3 (GAT-3) in the plasma membrane through downregulation of Rab11a. Huntington’s disease model astrocytes also showed reduced Ca^2+^ signaling and excessive self-grooming behaviors in a GAT-3 dependent manner, indicating that attenuation of Ca^2+^ signals by enhancing Ca^2+^ efflux through the plasma membrane affects the function of astrocytes, circuits and behaviors [109]. The effect of hPMCA2w/b on reducing astrocytic cytosolic Ca^2+^ was strong but smaller than that in IP_3_R2KO mice. However, IP_3_R2KO mice are not reported to display abnormal grooming behavior. Why is the grooming behavior of IP_3_R2KO mice normal? In conventional IP_3_R2KO mice, IP_3_R2 is deleted from astrocytes throughout the brain and at all times; therefore, compensatory mechanisms might maintain biological functions of astrocytes. A more intriguing possibility is that subcellular differences in Ca^2+^ dynamics are relevant to the functions of astrocyte Ca^2+^ signals. hPMCA2w/b mice have highly reduced Ca^2+^ near the plasma membrane, while IP_3_R2KO mice lack the major Ca^2+^ release pathway from the ER. Therefore, subcellular Ca^2+^ dynamics may differ between hPMCA2w/b astrocytes and IP_3_R2KO astrocytes, which may explain the difference in behavioral phenotypes and indicate functional diversity of Ca^2+^ signals in astrocytes. In theory, the methods described above can be applied to astrocytes in specific circuits rather than the entire brain. Such approaches may help to more specifically elucidate the role of Ca^2+^ signals in reactive astrocytes and may reveal the detailed mechanisms underlying aberrant Ca^2+^ signals in reactive astrocytes.

## 8. Conclusions

Reactive astrocytes display spatiotemporally dynamic Ca^2+^ signals. Advanced methods such as the use of GECIs, two-photon microscopy and novel transgenic approaches have revealed the molecular mechanisms of the dynamic features of Ca^2+^ signals. Although it is difficult to generalize on the role of aberrant Ca^2+^ signals in astrocytes considering the heterogeneity of reactive astrocytes, Ca^2+^ signals in reactive astrocytes clearly indicate certain disease states and disease severity. Reactive astrocytes showing enhanced Ca^2+^ signals probably acquire “gain of toxicity”, which plays deleterious roles in disease progression, even in the presymptomatic phase of disease. There are many mechanisms underlying aberrant Ca^2+^ signals in reactive astrocytes. Different disease models may share a similar mechanism, while other models of similar diseases may use distinct mechanisms. Some of these mechanisms are potentially targets to treat the disease. Why are so many different mechanisms involved in aberrant Ca^2+^ signals in astrocytes? We don’t have a clear answer for this right now. However, it may be a consequence of astrocyte heterogeneity in different circumstances (e.g., young vs. old, early phase vs. late phase, hippocampus vs. striatum); reactive astrocytes may show a broad and graded spectrum of molecular, cellular and functional changes [9] that produce distinct phenotypes depending on the stimuli [28].

To understand the functional implications of aberrant Ca^2+^ signals, experiments need to be stringently designed to take into account astrocyte heterogeneity in different circuits, and during development and aging. Even in the same experimental settings, astrocytes react immediately to brain insults to change their properties. To understand how Ca^2+^ signals are regulated in reactive astrocytes, it is therefore important to analyze astrocyte properties by non-biased methods for each experimental setting. This approach will allow us to understand not only the mechanisms underlying aberrant Ca^2+^ signals but also downstream signaling. In addition, it is important to develop a method to precisely manipulate Ca^2+^ signals in (patho-)physiological states without affecting unwanted targets [91]. A combination of the approaches described above may elucidate the role of reactive astrocytes in neurological diseases and help to find novel therapeutic targets to treat such diseases. Since astrocytes change their morphology and gene expression immediately in response to environmental changes [27,110], both in vitro and in vivo experiments are needed to understand the cellular functions of reactive astrocytes.

Ca^2+^ signal is one type of cellular activities. Other signals, such as cAMP and Na^+^, are also dynamically regulated in astrocytes. Improvement of Ca^2+^ imaging techniques has advanced our understanding of Ca^2+^ signals in astrocytes. Similarly, refinement of sensors for other signals will reveal novel insights into astrocyte activities enabling investigation of how distinct activities interact and cooperate in physiology and pathophysiology.

## Abbreviations

AD	Alzheimer’s disease
ALS	Amyotrophic lateral sclerosis
AM	Acetoxymethyl
AxCa	Aberrant extra-large Ca^2+^ signal
AxD	Alexander disease
CSD	Cortical spreading depression
DS	Down syndrome
EAE	Experimental autoimmune encephalitis
ER	Endoplasmic reticulum
GAT-3	GABA transporter 3
GECI	Genetically encoded Ca^2+^ indicator
GFAP	Glial fibrillary acidic protein
GqPCR	Gq-protein coupled receptor
hPMCA2w/b	Human PMCA2w/b
IP_3_R2	Inositol-1,4,5 trisphosphate receptor type2
IP_3_R2KO	IP_3_R type2 knockout
MCAO	Middle cerebral artery occlusion
mGluR5	Metabotropic glutamate receptor 5
MSN	Medium spiny neuron
mPTP	Mitochondrial permeability transition pore
MS	Multiple sclerosis
OGD	Oxygen glucose deprivation
PID	Peri-infarct depolarization
RTT	Rett syndrome
SCI	Spinal cord injury
SE	Status epilepticus
SIC	Slow inward current
TBI	Traumatic brain injury
TSP1	Thrombospondin 1
